# Supplementary material for: Shotgun Proteomics Revealed Preferential Degradation of Misfolded In Vivo Obligate GroE Substrates by Lon Protease in Escherichia coli
Source: Molecules. 2022 Jun 11;27(12):3772. doi: 10.3390/molecules27123772 (PMC9228906; doi:10.3390/molecules27123772)
Supplement: Supplementary file 1 [file molecules-27-03772-s001.zip › Supplementary Figures and Tables.pdf]

Supplementary materials of

Shotgun proteomics revealed preferential degradation of misfolded *in vivo* obligate GroE substrates by Lon protease in *Escherichia coli.*

Tatsuya Niwa, Yuhei Chadani, and Hideki Taguchi*

Cell Biology Center, Institute of Innovative Research, Tokyo Institute of Technology, Yokohama 226-8503, Japan

This document contains the following information:

· Supplementary Figure S1: Additional data of proteome analysis of MGM100 and MGM100-derived strains.
· Supplementary Figure S2: Additional data of proteome analysis of Δ*dnaKJ* and Δ*dnaKJ*Δ*lon* strains.
· Supplementary Table S1: Abundance changes of known Lon substrates in MGM100 and MGM100Δ*lon* strains.
· Supplementary Table S2: *E. coli* strains used in this study.
· Supplementary Table S3: Parameters for the SWATH acquisition.

Supplementary datasets are available as separated excel files:

· Supplementary Dataset S1 : Proteome data for the MGM100 and MGM100-derived strains.
· Supplementary Dataset S2 : Proteome data for the Δ*dnaKJ* and Δ*dnaKJ*Δ*lon* strains.
· Supplementary Dataset S3 : List of proteins with cluster numbers.
· Supplementary Dataset S4 : List of proteins up- or down-regulated by the Lon deletion.

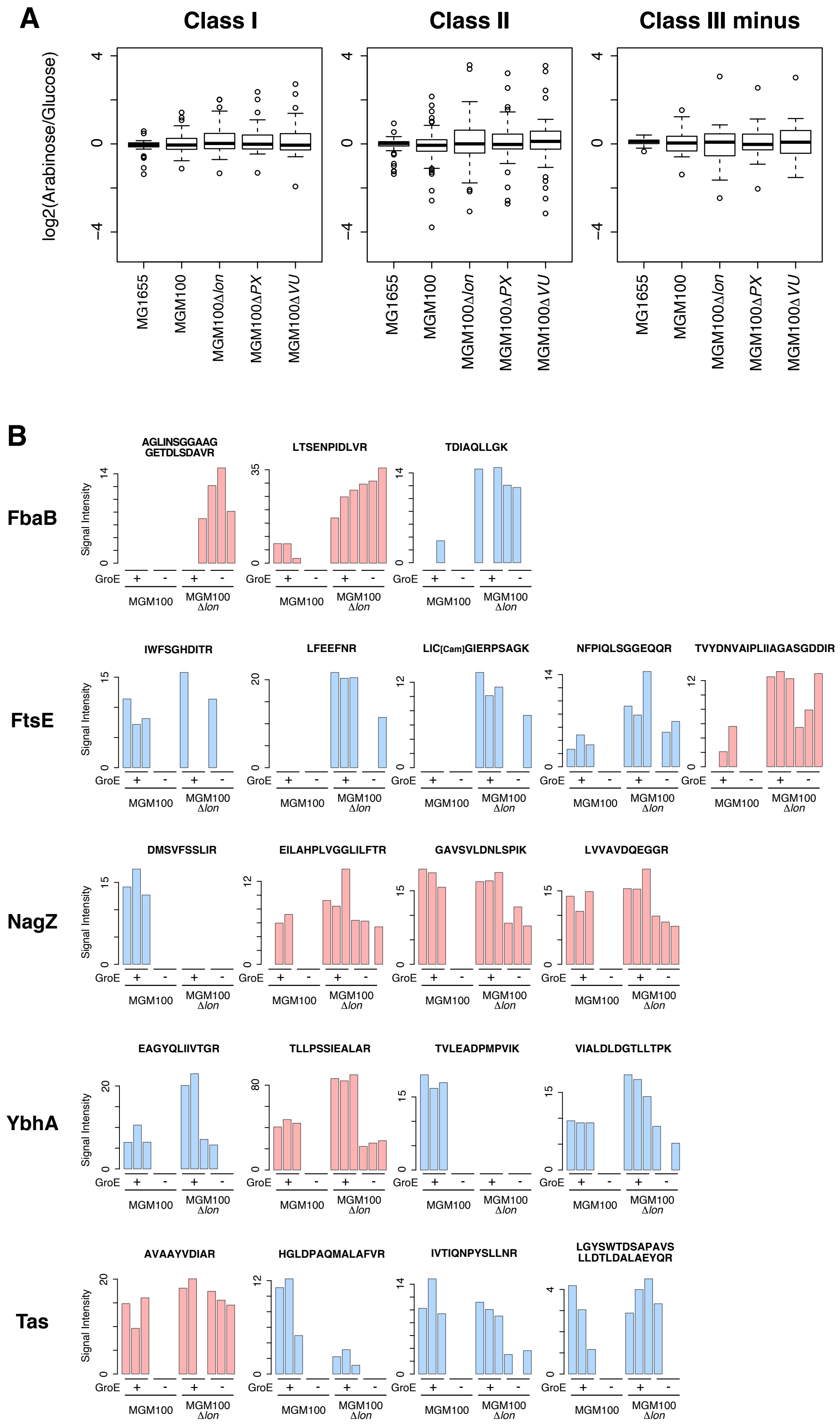

A
Class I
Class II
Class III minus
log2(Arabinose/Glucose)
MG1655
MGM100
MGM100Δlon
MGM100ΔPX
MGM100ΔVU
B
FbaB
FtsE
NagZ
YbhA
Tas
Signal Intensity
GroE
AGLINSGGAAG GETDLSDAVR
LTSENPIDLVR
TDIAQLLGK
IWFSGHDITR
LFEEFNR
LIC[Cam]GIERPSAGK
NFPIQLSGGEQQR
TVYDNVAIPLIIAGASGDDIR
DMSVFSSLIR
EILAHPLVGGLILFTR
GAVSVLDNLSPIK
LVVAVDQEGGR
EAGYQLIIVTGR
TLLPSSIEALAR
TVLEADPMPVIK
VIALDLDGTLLTPK
AVAAYVDIAR
HGLDPAQMALAFVR
IVTIQNPYSLLNR
LGYSWTDSAPAVS LLDTLDALAEYQR

Supplementary Figure S1. Additional data of proteome analysis of MGM100 and its derivative strains.

(A) Distribution of the foldchanges of GroE Class I, II, and III minus substrates represented as boxplots (by R software). The center bar in the box represents the median, and the upper and lower portion of the box represent 75% and 25% quantile, respectively. The numbers of the evaluated substrates were ~36 for Class I, ~77 for Class II, and ~29 for Class III minus.

(B) Intensities of the peptides corresponding to the five proteins identified in the GroE-normal cells but not in the GroE-depleted cells in the MGM100 strain (see Table 1). Only the intensities of MGM100 and MGM100Δ*lon* strains were shown. The graphs depicted in red indicate the peptides whose intensities under the GroE depletion condition in MGM100Δ*lon* were detected in all three technical replicates, *i.e.* the peptides assumed to be present under this condition.

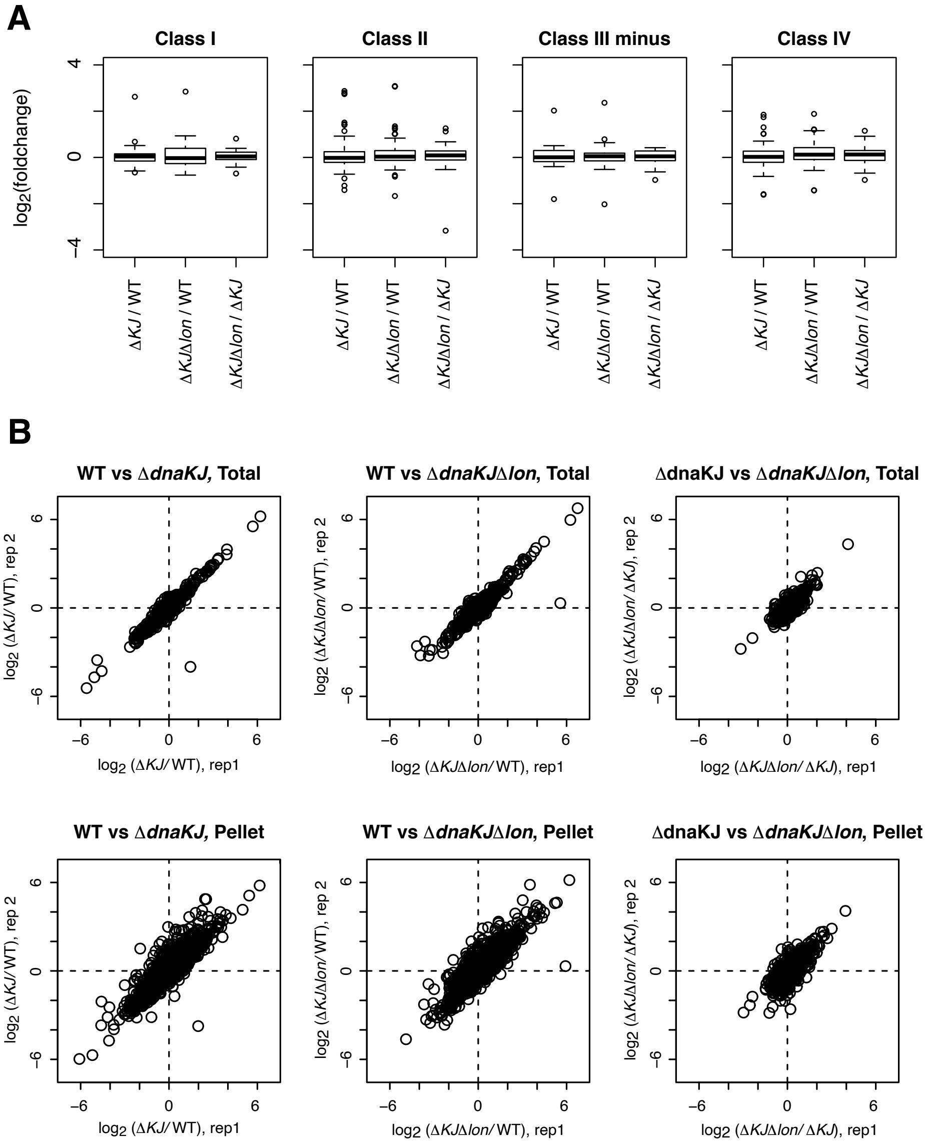

Supplementary Figure S2. Additional data of proteome analysis of Δ*dnaKJ* and Δ*dnaKJ*Δ*lon* strains.

(A) Distribution of the foldchanges of GroE Class I, II, III minus, and IV substrates represented as boxplots (by R software). The center bar in the box represents the median, and the upper and lower portion of the box represent 75% and 25% quantile, respectively. The numbers of the evaluated substrates were ~35 for Class I, ~81 for Class II, ~28 for Class III minus, and ~38 for Class IV.

(B) Reproducibility of the proteome changes by the DnaK/DnaJ deletion. Upper panels showed the reproducibility of the fold changes between two biological replicates in the total fraction, and lower panels showed those in the pellet fraction.

Supplementary Table S1. Abundance changes of known Lon substrates in MGM100 and MGM100Δ*lon* strains.

Protein Name	Gene name	Reference	Number of Detection MGM100 Ara	Number of Detection MGM100 Glc	Number of Detection MGM100 Δ*lon* Ara	Number of Detection MGM100 Δ*lon* Glc	Foldchange Glc/Ara, in MGM100	Foldchange Glc/Ara, in MGM100 Δ*lon*	Foldchange MGM100Δ*lon* / MGM100, Ara	Foldchange MGM100Δ*lon* / MGM100, Glc
SulA	*sulA*	*Proc. Natl. Acad. Sci. U S A.* 1983 [1]	0	0	0	0	(N. D.)	(N. D.)	(N. D.)	(N. D.)
RcsA	*rcsA*	*J. Bacteriol*. 1991 [2]	0	0	0	**3**	(N. D.)	(N. D.)	(N. D.)	(N. D.)
λXis	*xis*	*J. Bacteriol.* 1998 [3]	0	0	0	0	(N. D.)	(N. D.)	(N. D.)	(N. D.)
Homoserine succinyl transferase (HTS)	*metA*	*Mol. Microbiol.* 2000 [4]	1	**3**	2	**3**	(N. D.)	(N. D.)	(N. D.)	**2.96**
SoxS	*soxS*	*Mol. Microbiol.* 2004 [5]	0	0	0	0	(N. D.)	(N. D.)	(N. D.)	(N. D.)
MarA	*marA*		0	0	**3**	**3**	(N. D.)	**1.39**	(N. D.)	(N. D.)
RecA	*recA*	*Mol. Cell.* 2006 [6]	**3**	**3**	**3**	**3**	**1.15**	**0.98**	**1.54**	**1.32**
RuvB	*ruvB*		**3**	**3**	**3**	**3**	**0.84**	**1.34**	**1.48**	**2.36**
IbpA	*ibpA*	*Mol. Microbiol.* 2010 [7]	**3**	**3**	**3**	**3**	**2.51**	**2.59**	**1.81**	**1.86**
IbpB	*ibpB*		**3**	**3**	**3**	**3**	**1.62**	**2.65**	**1.3**	**2.13**
CspD	*cspD*	*Mol. Microbiol.* 2011 [8]	**3**	**3**	**3**	**3**	**0.69**	**0.4**	**1.03**	**0.59**
FdoH	*fdoH*	*J. Biol. Chem.* 2012 [9]	**3**	**3**	**3**	2	**0.62**	(N. D.)	**0.78**	(N. D.)
MetR	*metR*	*Proteomics*. 2018 [10]	1	**3**	0	**3**	(N. D.)	(N. D.)	(N. D.)	**1.27**
CysB	*cysB*		**3**	**3**	**3**	**3**	**0.95**	**1.47**	**0.98**	**1.52**
PuuB	*puuB*		0	0	0	0	(N. D.)	(N. D.)	(N. D.)	(N. D.)
YceA	*yceA*		**3**	**3**	**3**	**3**	**0.87**	(N. D.)	**0.64**	(N. D.)
FrsA	*frsA*		**3**	**3**	**3**	**3**	**1.29**	**1.06**	**1.57**	**1.29**
TldD	*tldD*		**3**	1	**3**	1	(N. D.)	(N. D.)	**1.68**	(N. D.)
**LipA***	***lipA***		**3**	**3**	**3**	**3**	**0.18**	**0.41**	**1.23**	**2.85**
SrlD	*srlD*		**3**	0	**3**	0	(N. D.)	(N. D.)	**3.77**	(N. D.)
YghW	*yghW*		0	0	0	0	(N. D.)	(N. D.)	(N. D.)	(N. D.)
NuoB	*nuoB*		**3**	2	**3**	**3**	(N. D.)	**0.16**	**0.85**	(N. D.)
FlgE	*flgE*		**3**	0	**3**	0	(N. D.)	(N. D.)	**0.73**	(N. D.)
ProV	*proV*		**3**	**3**	**3**	**3**	**1.17**	**0.71**	**0.91**	**0.55**
YrdA	*yrdA*		**3**	**3**	**3**	**3**	**1.47**	**1.14**	**1.08**	**0.84**
InaA	*inaA*		0	0	**3**	**3**	(N. D.)	**0.71**	(N. D.)	(N. D.)

* *In vivo* obligate GroE substrate

Supplementary Table S2. *E. coli* strains used in this study.

Strain	Description	Source
BW25113	F^-, λ^-, Δ(*araD*-*araB*)567, Δ*lacZ*4787(::*rrnB*-3), *rph*-1, Δ(*rhaD*-*rhaB*)568, *hsdR*514	Laboratory stock
JW0013-KC	BW25113 Δ*dnaK*::FRT-Km^r-FRT	Baba *et al.*, 2006 [11]
JW0014-KC	BW25113 Δ*dnaJ*::FRT-Km^r-FRT	
JW0427-KC	BW25113 Δ*clpP*::FRT-Km^r-FRT	
JW0428-KC	BW25113 Δ*clpX*::FRT-Km^r-FRT	
JW0429-KC	BW25113 Δ*lon*::FRT-Km^r-FRT	
JW3902-KC	BW25113 Δ*hslU*::FRT-Km^r-FRT	
JW3903-KC	BW25113 Δ*hslV*::FRT-Km^r-FRT	
ECY210	BW25113 Δ*lon*::FRT-Cm^r-FRT	This study
ECY262	BW25113 Δ*dnaKJ*::FRT-Km^r-FRT	
ECY289	BW25113 Δ*hslUV*::FRT-Km^r-FRT	
ECY290	BW25113 Δ*clpXP*::FRT-Km^r-FRT	
ECY292	BW25113 Δ*hslUV*::FRT-Cm^r-FRT	
ECY293	BW25113 Δ*clpXP*::FRT-Cm^r-FRT	
MGM100	F^-, λ^-, *rph-1, araBADp-groE*, *zje-2335*::*kan*	McLennan & Masters, 1999 [12]
MGM100Δ*lon*	MGM100 Δ*lon*::FRT-Cm^r-FRT	This study
MGM100Δ*clpXP*	MGM100 Δ*clpXP*::FRT-Cm^r-FRT	
MGM100Δ*hslUV*	MGM100 Δ*hslUV*::FRT-Cm^r-FRT	
MG1655	F^-, λ^-, *ilvG*$^-$, *rfb*-50, *rph*-1	Laboratory stock
MG1655Δ*dnaKJ*	MG1655 Δ*dnaKJ*::FRT-Km^r-FRT	This study
MG1655Δ*dnaKJ*Δ*lon*	MG1655 Δ*dnaKJ*::FRT-Km^r-FRT, Δ*lon:*:FRT-Cm^r-FRT	

Supplementary Table S3. Parameters for SWATH acquisition.

	TripleTOF 4600	TripleTOF 6600
Acetonitrile gradient<br>(in the presence of<br>0.1 % formic acid)	10 - 40 %, 67 min	10 - 30 %, 60 min<br>+<br>30 - 40 %, 10 min
m/z range of MS1	400 - 1250 Da	350 - 1500 Da
Accumulation time<br>for MS1	100 msec	100 msec
Accumulation time<br>for MS2	60 msec	45 msec
Number of SWATH<br>window	40	50
m/z of<br>SWATH window<br>(variable window<br>optimized for<br>*E. coli* cell lysate)	399.5 - 424.2<br>423.2 - 445.1<br>444.1 - 462.1<br>461.1 - 477.5<br>476.5 - 491.8<br>490.8 - 505.6<br>504.6 - 518.2<br>517.2 - 530.9<br>529.9 – 543.0<br>542.0 - 553.9<br>552.9 - 564.9<br>563.9 - 575.9<br>574.9 - 585.8<br>584.8 - 596.3<br>595.3 - 606.7<br>605.7 - 616.6<br>615.6 - 627.1<br>626.1 – 637.0<br>636.0 - 646.9<br>645.9 - 657.3<br>656.3 - 667.8<br>666.8 - 678.2<br>677.2 - 688.1<br>687.1 - 699.1<br>698.1 - 709.6<br>708.6 - 720.6<br>719.6 - 732.1<br>731.1 - 744.2<br>743.2 - 756.9<br>755.9 - 770.1<br>769.1 - 784.4<br>783.4 - 800.3	349.5 - 378.7<br>377.7 - 405.7<br>404.7 - 425.8<br>424.8 - 441.9<br>440.9 - 456.3<br>455.3 - 470.1<br>469.1 - 482.7<br>481.7 - 494.2<br>493.2 - 505.7<br>504.7 - 516.7<br>515.7 - 527.0<br>526.0 - 537.4<br>536.4 - 547.2<br>546.2 - 556.4<br>555.4 - 565.6<br>564.6 - 574.8<br>573.8 - 583.4<br>582.4 - 592.0<br>591.0 - 600.6<br>599.6 - 609.3<br>608.3 - 617.9<br>616.9 - 627.1<br>626.1 - 635.7<br>634.7 - 644.9<br>643.9 - 654.1<br>653.1 - 663.9<br>662.9 - 674.2<br>673.2 - 684.0<br>683.0 - 694.4<br>693.4 - 705.3<br>704.3 - 716.2<br>715.2 - 728.3

	799.3 - 816.8<br>815.8 – 835.0<br>834.0 - 854.8<br>853.8 - 876.8<br>875.8 - 901.5<br>900.5 - 929.6<br>928.6 - 960.9<br>959.9 - 999.4	727.3 - 740.4<br>739.4 - 753.0<br>752.0 - 766.2<br>765.2 - 780.6<br>779.6 - 796.1<br>795.1 - 812.2<br>811.2 - 829.5<br>828.5 - 847.9<br>846.9 - 867.4<br>866.4 - 887.6<br>886.6 - 908.8<br>907.8 - 932.4<br>931.4 - 958.3<br>957.3 - 987.0<br>986.0 - 1022.1<br>1021.1 - 1070.4<br>1069.4 - 1126.2<br>1125.2 - 1199.8

References

1. Mizusawa, S.; Gottesman, S. Protein Degradation in *Escherichia Coli*: The Lon Gene Controls the Stability of SulA Protein. *Proc Natl Acad Sci U S A* **1983**, *80*, 358–362, doi:10.1073/PNAS.80.2.358.
2. Stout, V.; Torres-Cabassa, A.; Maurizi, M.R.; Gutnick, D.; Gottesman, S. RcsA, an Unstable Positive Regulator of Capsular Polysaccharide Synthesis. *J Bacteriol* **1991**, *173*, 1738–1747, doi:10.1128/JB.173.5.1738-1747.1991.
3. Leffers, J.; Gottesman, S. Lambda Xis Degradation in Vivo by Lon and FtsH. *J Bacteriol* **1998**, *180*, 1573–1577, doi:10.1128/JB.180.6.1573-1577.1998.
4. Biran, D.; Gur, E.; Gollan, L.; Ron, E.Z. Control of Methionine Biosynthesis in *Escherichia Coli* by Proteolysis. *Mol Microbiol* **2000**, *37*, 1436–1443, doi:10.1046/J.1365-2958.2000.02097.X.
5. Griffith, K.L.; Shah, I.M.; Wolf, R.E. Proteolytic Degradation of *Escherichia Coli* Transcription Activators SoxS and MarA as the Mechanism for Reversing the Induction of the Superoxide (SoxRS) and Multiple Antibiotic Resistance (Mar) Regulons. *Mol Microbiol* **2004**, *51*, 1801–1816, doi:10.1046/J.1365-2958.2003.03952.X.
6. Neher, S.B.; Villén, J.; Oakes, E.C.; Bakalarski, C.E.; Sauer, R.T.; Gygi, S.P.; Baker, T.A. Proteomic Profiling of ClpXP Substrates after DNA Damage Reveals Extensive Instability within SOS Regulon. *Mol Cell* **2006**, *22*, 193–204, doi:10.1016/J.MOLCEL.2006.03.007.
7. Bissonnette, S.A.; Rivera-Rivera, I.; Sauer, R.T.; Baker, T.A. The IbpA and IbpB Small Heat-Shock Proteins Are Substrates of the AAA+ Lon Protease. *Mol Microbiol* **2010**, *75*, 1539–1549, doi:10.1111/J.1365-2958.2010.07070.X.
8. Langklotz, S.; Narberhaus, F. The *Escherichia Coli* Replication Inhibitor CspD Is Subject to Growth-Regulated Degradation by the Lon Protease. *Mol Microbiol* **2011**, *80*, 1313–1325, doi:10.1111/J.1365-2958.2011.07646.X.
9. Westphal, K.; Langklotz, S.; Thomanek, N.; Narberhaus, F. A Trapping Approach Reveals Novel Substrates and Physiological Functions of the Essential Protease FtsH in *Escherichia Coli*. *J Biol Chem* **2012**, *287*, 42962–42971, doi:10.1074/JBC.M112.388470.
10. Arends, J.; Griego, M.; Thomanek, N.; Lindemann, C.; Kutscher, B.; Meyer, H.E.; Narberhaus, F. An Integrated Proteomic Approach Uncovers Novel Substrates and Functions of the Lon Protease in *Escherichia Coli*. *Proteomics* **2018**, *18*, doi:10.1002/PMIC.201800080.
11. Baba, T.; Ara, T.; Hasegawa, M.; Takai, Y.; Okumura, Y.; Baba, M.; Datsenko, K.A.; Tomita, M.; Wanner, B.L.; Mori, H. Construction of *Escherichia Coli* K-12 in-Frame, Single-Gene Knockout Mutants: The Keio Collection. *Mol Syst Biol* **2006**, *2*, doi:10.1038/MSB4100050.
12. McLennan, N.; Masters, M. GroE Is Vital for Cell-Wall Synthesis. *Nature* **1998**, *392*, 139, doi:10.1038/32317.
